# Teaching laboratory for large cohorts of undergraduates: Private and social information in fish

**DOI:** 10.1002/ece3.5889

**Published:** 2019-12-02

**Authors:** Jost Borcherding, Mike M. Webster, Katja Heubel

**Affiliations:** ^1^ Institute for Zoology, General Ecology Ecological Research Station Rees University of Cologne Cologne Germany; ^2^ School of Biology University of St Andrews St Andrews UK; ^3^ Research and Technology Centre (FTZ) Kiel University Büsum Germany

**Keywords:** behavior, feeding, *Gasterosteus aculeatus*, learning, trade‐off

## Abstract

A challenge in the Bachelor's studies in Biology is to strike a balance between reducing the teaching of practical scientific experiments to what is feasible in a short time, and teaching “real” science in undergraduate laboratories for high numbers of participants. We describe a laboratory in behavioral biology, with the primary focus on the student learning. However, also the underlying scientific question and the results of the experiment, namely the behavior of the three‐spined stickleback (*Gasterosteus aculeatus*) in a trade‐off situation during foraging, is without a doubt timely and sufficient for scientific studies on this subject, and this through the experiments conducted and data collected by the students. The students rated this laboratory well and learned at the end that social information is certainly important, but that self‐learning can be more important, and this not only in small fish, but also for the students themselves.

## Graphical abstract

We describe a laboratory in behavioral biology, with the primary focus on the student learning. However, also the underlying scientific question and the results of the experiment, namely the behavior of the three‐spined stickleback (*Gasterosteus aculeatus*) in a trade‐off situation during foraging, are without a doubt timely and sufficient for scientific studies on this subject, and this through the experiments conducted and data collected by the students. The students rated this laboratory well.

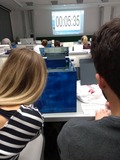

## 1 INTRODUCTION AND TASK DEFINITION

The education of students in the Bachelor's studies in Biology is a balancing act between the reduction of scientific experiments to what is feasible in a short time, and the challenge to teach “real” science in undergraduate laboratories (Wilson, 2013), considering the often high number of participants. This is a particular challenge in behavioral research when experiments with living animals need to be feasible and tangible for all laboratory participants (Angra, Weigel, & Onstine, 2018; Tanner & Allen, 2004). In addition to teaching concepts and practical skills, laboratories should encourage students to think about and take responsibility for the ethical treatment of living beings in experiments (Randler, Binngießer, & Vollmer, 2019). Apart from supporting retention and mitigating dislike or fear of unfamiliar animals (Bauerle & Park, 2012; Dohn, Madsen, & Malte, 2009; Randler, Hummel, & Prokop, 2012), such real encounters may provide a basis for educating students to become responsibly acting and critically thinking people, which is fundamentally relevant not only for studies in biology, but for the understanding to protect our planet. For this purpose, we designed an experiment to familiarize students with concepts and tasks of studying animal behavior by addressing a scientifically relevant and well‐studied question (Brown & Laland, 2003). Specifically, students test three‐spined sticklebacks (*Gasterosteus aculeatus*), for their ability (a) to learn the usage of a novel feeding site, (b) to utilize social information on an unknown feeding place (Coolen, van Bergen, Day, & Laland, 2003) and finally (c) whether there is a trade‐off between private and public information (van Bergen, Coolen, & Laland, 2004). This trade‐off and observers benefitting from utilizing the knowledge of informed observers still remains a puzzle (Roy & Bhat, 2017; Webster & Laland, 2018), and apart from developing a half‐day laboratory that can educate and provide larger groups of students with unique hands‐on experience on animal behavior, such laboratory exercises may also help us to collect valuable data sets and scientific insights. Since 2010, the laboratory took place with about 200–240 undergraduate students per year; each laboratory is divided into four courses (regularly around 50 students per course, up to 60 students are possible in this class room) that are then given in a row. Students evaluated all aspects of the laboratory positively, and the practicability of the laboratory in the short time available was proven. We encourage academic teachers to conduct similar laboratories.

## 2 SETUP OF THE LABORATORY

The laboratory (4 hr) on the behavior of small, short‐lived locally available fish, in this case, the three‐spined stickleback, as part of the Basic Module “Ecology” within the 2nd year bachelor studies in Biological Sciences at the University of Cologne, Germany, is designed for 200–240 students. In preparation for this laboratory, about 200 sticklebacks are acquired each year (sticklebacks and other small native fish can be obtained in Germany e.g., from sellers for pond fish, in the UK from commercial suppliers) approx. 8 weeks before the start of the laboratory and acclimatized in the holding aquariums. Instead of sticklebacks, this practical could also be run with other commonly available species like zebrafish or guppies (e.g., Trompf & Brown, 2014), of which at least the latter are regularly available from laboratory stocks or commercial sellers. Nevertheless, with the appropriate organisms and knowledge at hand, the laboratory could be adjusted to work with invertebrates such as ants (Czaczkes, Beckwith, Horsch, & Hartig, 2019; Oberhauser, Schlemm, Wendt, & Czaczkes, 2019), drosophila (Mery et al., 2009), crickets (Coolen, Dangles, & Casas, 2005), and fiddler crabs (Angra et al., 2018) that are already proven to use social information and are available and testable in an appropriate experimental setup.

**Figure 1:**
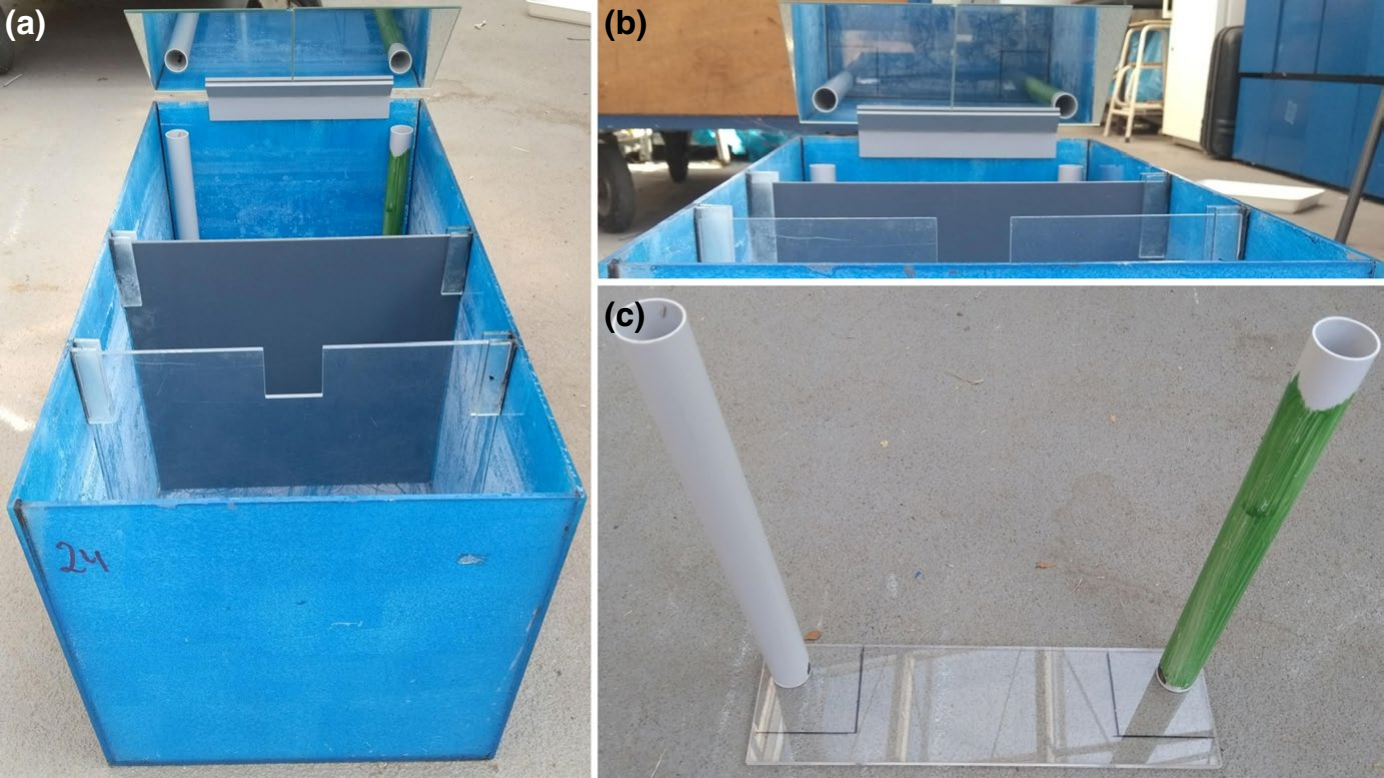
Picture of one experimental tank as it is used by the students to observe the behavior of sticklebacks, including the two dividing plates, the green and gray feeding tubes fixed on a Plexiglas base plate, and the mirror attached to the tank in a 45° angle. (a) Fully equipped tank; (b) the student's view through the mirror on the observation area; (c) detailed view on the feeding tubes put on the Plexiglas base plate

The animals are trained or familiarized to the experimental conditions at least 30 times for about 6 weeks (Appendix S1). For the laboratory, 24 aquaria are prepared in a course room. Each aquarium is equipped with various utensils for the experimental setup (Figure 1). The one‐time costs for this setup amount to around 2,500–3,000 €; in addition, there are annual costs of around 150–200 € for the purchase of the sticklebacks and frozen food. The laboratory was then held four times with 50–60 students per laboratory within three days, with groups of 2–3 students carrying out a complete hands‐on behavior test using live sticklebacks (Appendix S2). With the proposed test design, 12 replicates of the behavior tests are thus carried out each year. The actual experimental fish, the two groups of observers (ObsTrained, ObsUntrained), are of course only used once in this setup to avoid pseudoreplication. On the other hand, the demonstrators are used repeatedly, randomly chosen from holding tanks and assigned to test tanks in this setup. For these fish, the test period is just an extension of the training period as there is absolutely no difference between their training period and the current experimental conditions (cf. Appendix S1). The typical sequence of the four‐hour laboratory is roughly divided into Welcome/Administration/Attestation (20 min), introduction to the systematics and behavioral biology of fish (30 min), detailed explanation of the experiment and ethical handling of fish (40 min, which is also explained in the preparation script for the students that is made available as essential preparation for the course, Appendix S2; Magnhagen, Braithwaite, Forsgren, & Kapoor, 2008), setup of the experiment by the students (20 min), execution of the experiment (30 min), cleaning of the test tanks and equipment as well as simultaneous data entry into the prepared data sheet (30 min), and finally discussion and interpretation of the results (60 min).

The extent to which such experiments and the use of live animals in teaching require permission certainly varies from country to country. In our case, we initially applied for an animal experiment in accordance with German legislation (based on EU legislation, European Union, 2010). In the evaluation by the responsible institutions in North Rhine‐Westphalia (LANUV NRW), it was decided that our behavioral experiments with the sticklebacks do not pose any risk of causing pain, suffering, or harm and thereby are not animal experiments in the sense of the legislation (approval number 81‐02.05.40.18.071).

The student evaluation questionnaire was approved by the appropriate university authorities, and completion of the questionnaire was both voluntary and anonymous for students. Under German regulations, use of this survey data in research such as this does not require separate ethical approval.

## 3 RESULTS AND DISCUSSION

### 3.1 Behavior of three‐spined sticklebacks

**Figure 2:**
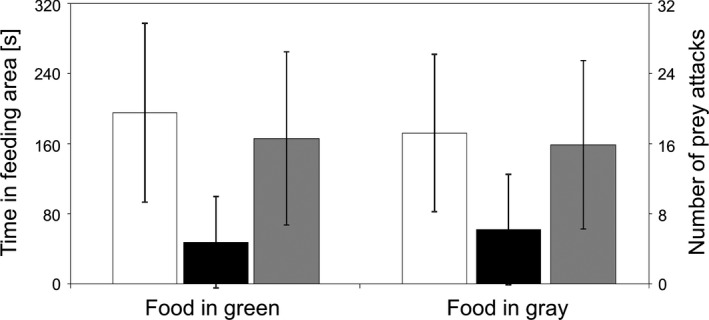
Performance of Demonstrator: No significant differences for all three factors between the setup when food was offered in the green tube or in the gray tube White = time in feeding area with food. Black = time in feeding area without food. Gray = number of prey attacks (all values mean ± *SD*, *t* test to compare one group with another [as it is to discuss with the students]: all *p* > .05)

Within the experiment, the feeding behavior of the “demonstrators” provides public information for the actual test fish, the “observers.” In addition, the demonstrators are used to account for potential biases of the test factors on the behavior of the test fish (color and position of the feeding tube). The results of this analysis of the potential biases showed very similar tendencies every year and, thus, allow the statement that neither the color of the feeding tube nor its position in the aquarium has influenced the feeding behavior of three‐spined sticklebacks (Chi^2^ test on a null expectation of 50:50; Table 1). This also applies to the most important potential bias: The experimental fish do not know where the food is (Table 1). Usually, these individual statistical analyses for each year are not significant compared to an expected 50:50 distribution (except single false‐positive results in certain years, Table 1). However, this of course clearly depends on the number of replicates conducted. For this reason, we always have a complete data set (or a mean value from several years) ready for discussion, especially for the first two rounds of the laboratory each year, that is when only three or six replicates were so far conducted by the students. As the number of replicates increased (over the laboratory each year and also over the years), smaller differences were also tested as significant by the test (cf. calculation of the Chi^2^ test for all values from 2010 to 2018, Table 1). In this way, we use these data in the laboratory as a good example to demonstrate the influence of effect size, number of replicates, what is a pseudoreplicate, etc. to the students (this also applies for all other statistics discussed with the students and presented here). All statistics presented here are only really basic statistics as they are discussed with the undergraduate students in the laboratory (we are aware that in most cases further analysis could be done with multivariate statistics, which is, however, not the primary focus of this presentation). Nevertheless, the specific task of the demonstrators, to provide public information for the test fish, is reliably fulfilled every year, with a significant longer stay in the feeding area with food and with their apparent feeding activity there (Figure 2).

**Table 1:** First choice of the demonstrators (in percent) that approached the feeding area, depending on the experimental factors, which were randomly applied in the tanks (color and position of the feeding tube, feeding tube with or without food)

	*n*	Factor color			Factor position			Factor food		
		Green	Gray	*p*	Right	Left	*p*	Yes	No	*p*
2010	95	56.8	43.2	.365	70.4	29.6	**.0056** a	60.2	39.8	.173
2011	88	56.5	43.5	.375	52.2	47.8	.768	63.0	37.0	.075
2012	92	56.8	43.2	.344	61.1	38.9	.125	52.6	47.4	.717
2013	87	54.0	46.0	.595	72.4	27.6	**.0024**	54.0	46.0	.595
2014	90	58.9	41.1	.231	58.9	41.1	.231	60.0	40.0	.178
2015	95	62.1	37.9	.093	48.4	51.6	.828	47.4	52.6	.717
2016	92	75.0	25.0	**.0005**	55.4	44.6	.46	62.0	38.0	.102
2017^b^	73	64.8	35.2	.075	52.1	47.9	.801	69.0	31.0	**.021**
2018	96	57.3	42.7	.311	46.9	53.1	.665	58.3	41.7	.247
All years	808	59.4	40.6	**.0002**	56.6	43.4	**.0083**	57.9	42.1	**.0014**

Note

The values are given for each year separately and as a calculation including all values over the period from 2010 to 2018. *n*, Number of replicates; *p*, Chi^2^ test comparing the experimental data with an expected 50:50 distribution, with significant values < .05 printed in bold.

a First experiments in a new room; unrecognized bias due to nondarkened windows.

b One of the four courses did not take place.

**Figure 3:**
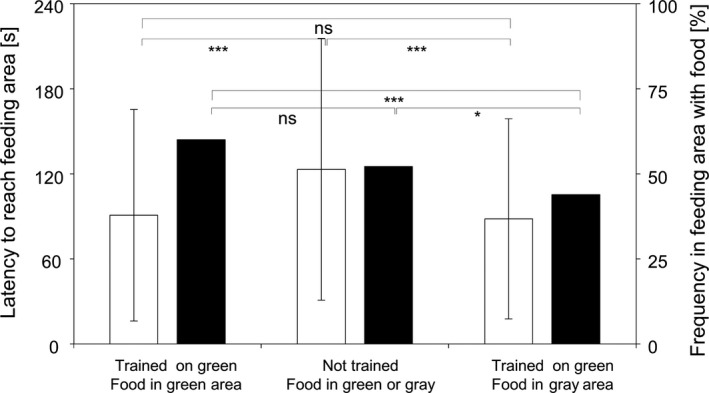
First reaction of Observer: There were no significant differences for the different groups of observers to reach one of the feeding areas (white, independently on where food was offered, all values mean ± *SD*). For those, observers were private and public information agreed the frequency to first reach in the feeding area with food (black) was significantly higher than for untrained fish and when there was a conflict between private and public information. (*n* = 781 trials; *t* test to compare one group with another [as it is to discuss with the students], ns = *p *> .05, **p < .01, ***p < .001)

**Figure 4:**
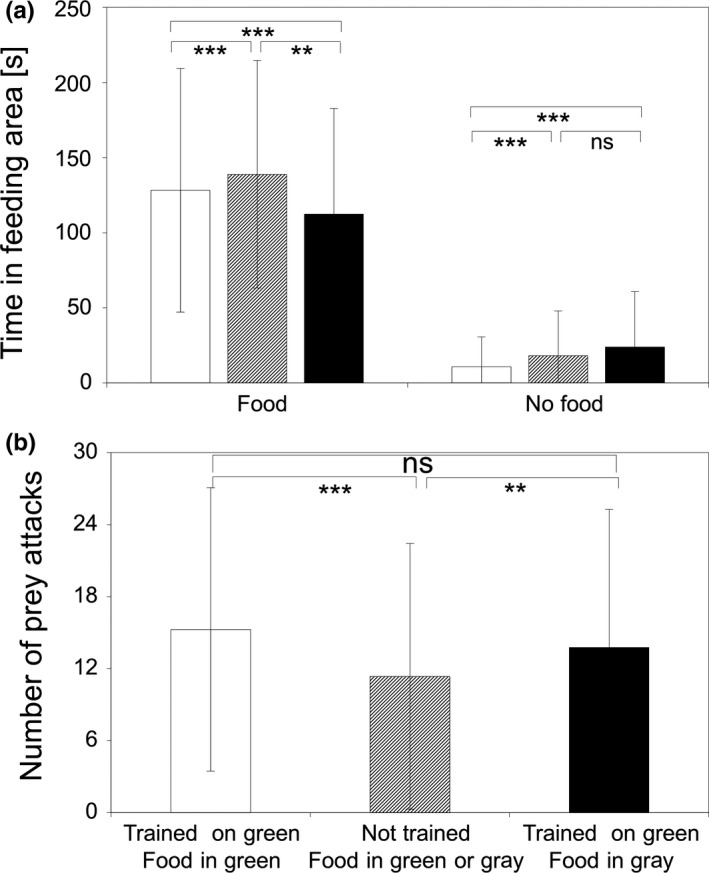
Performance of Observer: white = trained observers for which private and public information equal; striped = untrained observers; black = trained observers for which private and public information conflict. (a) Untrained observers increased their time in the area with food, and observers for which private and public information equal, reduced their time in the area without food (all values mean ± *SD*, *n* = 777 trials; *t* test to compare one group with another [as it is to discuss with the students], ns = *p *> .05, *p < .05, ***p < .001). (b) Number of prey attacks was highest for trained observers, independently if their private and public information equal or conflict, and lowest for untrained observers (all values mean ± *SD*, *n* = 777 trials; *t* test to compare one group with another [as it is to discuss with the students], ns = *p *> .05, **p < .01, ***p < .001)

Two groups of experimental observers are individually tested in the proposed test design: Observers that are trained to get their food at the green feeding tube (ObsTrained) and a control group (ObsUntrained) that also was familiarized to the experimental conditions at least 30 times during the pre‐experimental period, but never saw one of the feeding tubes and received their food only from the surface in their holding tanks. Observers for which private information match with just received public information (ObsTrained: trained on green feeding tube and food in green feeding tube) reached the feeding area more often on their first attempt than those for which there was a conflict between public and private information (ObsTrained: trained on green feeding tube and food in gray feeding tube). Untrained fish (ObsUntrained) were nearly as good in first reaching the right feeding area; however, they needed significantly more time than both experimental groups with trained fish (Figure 3). Though untrained fish increased their time in the feeding area where food was offered, they fed significantly less than both groups of trained sticklebacks, irrespective of whether the food was offered in the green or gray feeding tube (Figure 4). These results clearly support that three‐spined sticklebacks (a) can learn the usage of a new feeding place, (b) can make use of public/social information, and (c) that there is a potential trade‐off situation for those fish, for which there was a mismatch between private and public information.

These findings are consistent with those obtained in other studies of social foraging in three‐spined sticklebacks and related species (reviewed by Laland, Atton, & Webster, 2011). For example, in an experiment where three‐spined sticklebacks were allowed to forage over patches of sand or gravel, observers tended to forage on the same substrate types as demonstrators (Webster & Hart, 2006). They did this when they had no previous experience of finding food on either substrate type but also when they were experienced but were presented with conflicting public information (i.e., sand‐trained fish that were exposed to demonstrators feeding from gravel but not from sand also spent more time searching for food on the gravel, overriding their prior bias for foraging more on the sand). The cues provided by other animals as they forage provide observers with reliable information about the presence of food. Many animals, including sticklebacks, are sensitive to the feeding behavior of others, and readily approach conspecifics displaying these behaviors. In the case of sticklebacks such feeding cues include adopting head‐down posture and performing lunges or rapid strikes toward the substrate, behaviors which attract others even when food is not present (Riddell & Webster, 2017). By paying attention to these cues, foragers are able to exploit the discoveries of others, receiving cheap information as to the location of food, allowing them to minimize the time and energetic costs of searching for food themselves. These cues are likely by‐products of foraging behavior rather than signals to the observers, and for the demonstrators, attracting others to their feeding location may often be costly, since these compete for the available food. The dynamic costs and benefits of searching for food directly versus using cues from others to locate it indirectly are captured by the producer–scrounger family of social foraging models (Barnard & Sibly, 1981; Giraldeau & Caraco, 2000).

### 3.2 Learning objectives and students' experiences/evaluations

**Figure 5:**
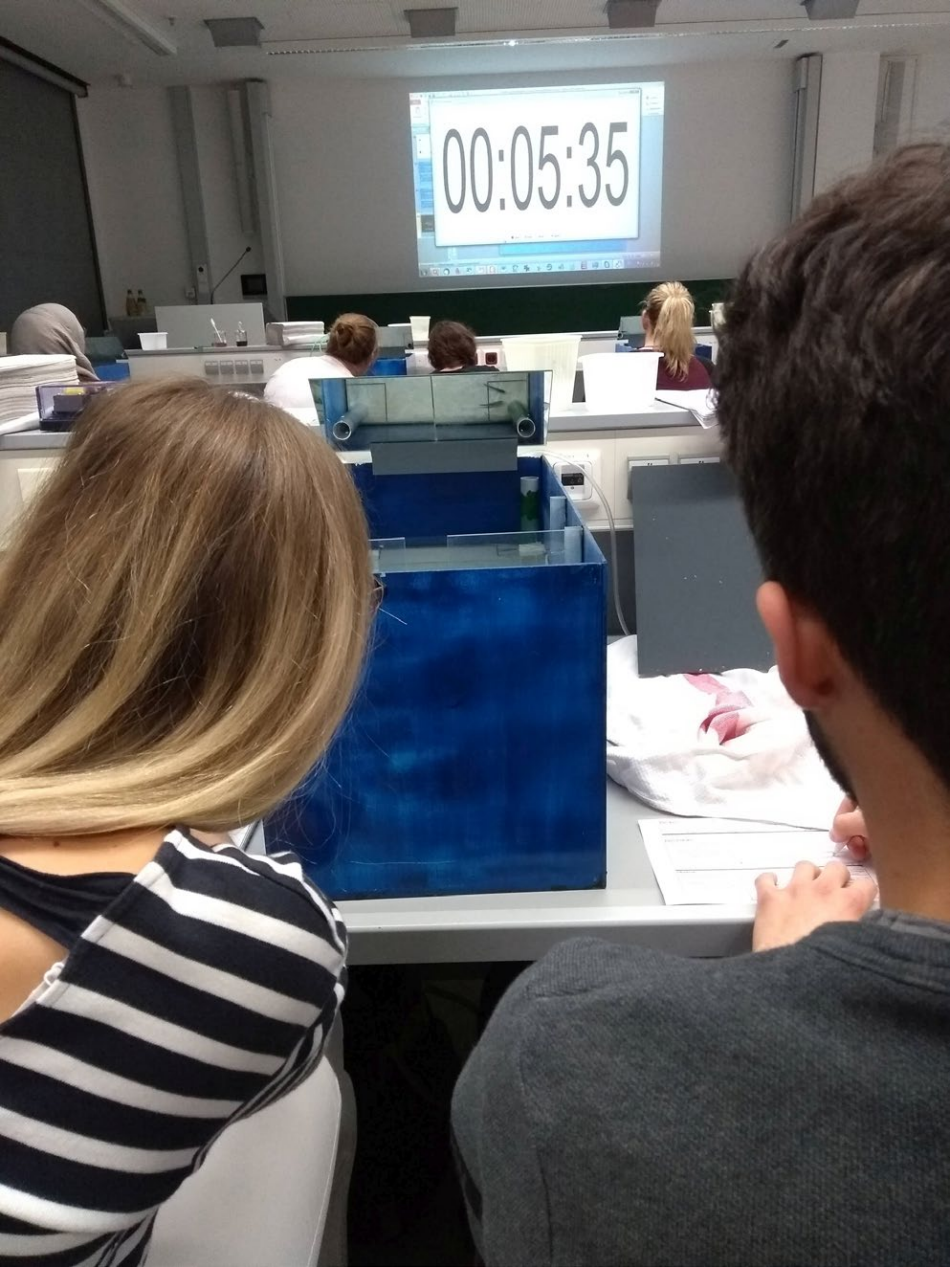
Students during the observational period to record stickleback behavior. From the students' point of view in the mirror, the two sticklebacks can be easily observed while feeding, without any disturbances occurring above the aquarium. The stopwatch is projected for the students and serves to synchronize the experiments

“All knowledge of reality starts from experience and ends in it” (Einstein, 1954), thus, best learning is to provide students the opportunity to gather own authentic practice/experience (Figure 5, Burrowes, 2003). Therefore, the laboratory was designed to cover the following more general aspects: (a) Formulating an hypothesis that can then be tested statistically. (b) Usage of Excel and basic statistics on the own collected data (including strategies to control for errors during data input, mistakes in measurements etc.; the Excel data file including all statistics Appendix S3). (c) Control and inference: what is an experimental bias and how can we omit these in behavioral experiments. The development and implementation of a rigorous design for such behavioral experiments, which allows results to be independent of potential biases, is one of the essential learning goals of this laboratory. Furthermore, the results provide the take‐home message for the students that learning and the function of a social environment are of great importance for skill acquirement.

In 2016 and 2017, one part of the students (the first of the four replicate laboratory groups each year) was asked to evaluate the laboratory (Dehne, 2017; Schmidt, 2016). The results for both years were nearly identical concerning the evaluation of the “fish behavior” laboratory within the Basic Module “Ecology” of the Bachelor studies in Biological Sciences, and are here partly (only those aspects which are primarily independent of the performance of the lecturer) presented using the 2017's data (Table 2). The results of this assessment clearly reveal that all aspects of the laboratory were evaluated positively, in particular the interest of the students was addressed and the practicability of the laboratory in the short time available was proven. This is underpinned by the personal experience of the lecturer (JB) that nearly after each laboratory, students directly stated that this laboratory had been the most interesting and best in their studies in biology so far.

**Table 2:** Partial results of the evaluation of the different laboratories (*n* = 13) within the Basic Course “Ecology,” as part of the laboratories of studies of the Bachelor in Biological Sciences at the University of Cologne (Dehne, 2017)

Question to students	Rating laboratory “fish behavior”	Mean rating all laboratories	Rating worst laboratory
The tasks are interesting	1.84 ± 0.89	2.92 ± 1.28	3.98 ± 0.97
The tasks contribute to my understanding of ecology	1.82 ± 0.92	2.87 ± 1.27	3.82 ± 1.23
The tasks are well practicable within the exercise	1.30 ± 0.51	2.52 ± 1.27	3.51 ± 1.27
The task is understandable	1.30 ± 0.51	2.12 ± 1.14	3.06 ± 1.24
Today's topic was interesting	1.52 ± 0.66	2.72 ± 1.30	3.75 ± 1.08
In today's exercise I learned a lot	2.14 ± 0.97	3.18 ± 1.22	4.02 ± 1.11
It made sense to carry out this exercise	1.93 ± 0.87	2.88 ± 1.28	3.82 ± 1.15
The transferability to other topics is given	2.05 ± 1.06	2.99 ± 1.32	3.77 ± 1.38

Note

In this survey, which was approved by the responsible staff of the university and of course absolutely voluntary as well as anonymous for the students, only the participants of the first course took part, which was held four times in a row. Mean rating score of the laboratory on “fish behavior” (ranked no 1 for all shown questions), mean rating for all laboratories and the rating of the laboratory with the lowest values are given. Possible answers of the students to the questions could vary between 1 (fully true) and 5 (does not apply at all); the results are given as mean ± *SD* (mean of 41.9 students, ≈90%, answering per laboratory, minimum 38, and in maximum 47, which equals the number of all participants in this particular course in 2017). Only questions from the evaluation sheet are shown that rate the laboratory topic and primarily not the lecturer's performance.

## 4 CONCLUSION

“Only a fool makes no experiments” as Charles Darwin said, however, to give a mass laboratory on animal behavior on a relevant research question with reliable outcome is quite a challenge. Behavior is one topic in zoology that is often skipped in basic courses because there are conflicting constrains of behavioral experiments, costs and the short time available for the laboratory. Nevertheless, motivation and learning can be deepened via Aha moments and hands‐on experiences with live animals (Dohn et al., 2009; Holstermann, Grube, & Bogeholz, 2010). Here, we provide the full setup for such a laboratory, the practicability of which has been demonstrated year after year, for numbers up to 240 students. All documents are available as Appendix [Link], [Link] with complete operating instructions for the training of sticklebacks (to achieve reliable private information) and the experimental setup as well as instructions for students, including analyses of results with an automated Excel sheet that also addresses graphical presentation and statistics (Borcherding, Webster, & Heubel, 2019). To conduct such an experiment and the subsequent discussion of the hypotheses and results allows the evaluation of an appropriate experimental design and how potential biases can be assessed not to be effective. This laboratory is an example of how to deal with constrains of a mass event, that allows the students to conduct all steps of a behavioral experiment within a short time span, achieving own practical experiences.

## CONFLICT OF INTEREST

The authors declare no conflict of interest.

## AUTHOR CONTRIBUTIONS

J.B. designed the laboratory and taught the course at the University of Cologne from 2010 onwards. All data were sampled by numerous students. Analysis of the behavioral data and their interpretation was conducted by M.M.W. and J.B. K.H. conceptionalized the dissemination of this academic practise. All authors wrote the manuscript.

## Data Availability

Operating instructions for the training of sticklebacks (to achieve reliable private information), the experimental setup as well as instructions for students, including analyses of results with an automated Excel sheet that also addresses graphical presentation and statistics and the data for stickleback behavior will be accessible as supplemental material (https://doi.org/10.5061/dryad.tx95x69sh; Borcherding et al., 2019).
