# Supplementary material for: Teaching laboratory for large cohorts of undergraduates: Private and social information in fish
Source: Ecol Evol. 2019 Dec 2;10(1):4–10. doi: 10.1002/ece3.5889 (PMC6972818; doi:10.1002/ece3.5889)
Supplement: Supplementary file 1 [file ECE3-10-4-s001.docx]

Supplementary material to

Teaching lab for large cohorts of undergraduates: private and social information in fish

Jost Borcherding, Mike M. Webster and Katja Heubel

S1 Training of sticklebacks – operating instruction for teachers and tutors

S2 Experiments with sticklebacks by students – preparation script for students

S3 Analysis of stickleback experiments – Excel-sheet (in a separate Supplement file)

S1 Training of sticklebacks – operating instruction for teachers and tutors

**The role of social learning and personal experience in threespined stickleback, *Gasterosteus aculeatus*, foraging behaviour**


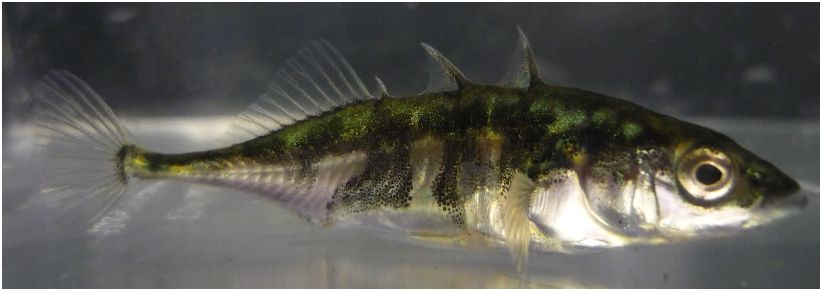


**Holding and Training**

**Holding**

Approx. 200 sticklebacks are required for the experiment. Among them are 69 fish as demonstrators, and 132 as observers. The observers are divided into 2 groups, with 66 trained on the green feeding tube (ObsTrained) and 66 inexperienced sticklebacks (ObsUntrained). 9 aquariums are used to hold these fish. 3 tanks are occupied by 22 untrained sticklebacks each, 3 with 22 sticklebacks trained on the green feeding tube each and 3 tanks with 23 demonstrators each. The sticklebacks are kept in their aquariums for at least five days and fed twice a day with some defrosted Chironomid larvae to keep the hunger level constant throughout the entire experimental period.

**Training**

Two aquariums with a size of 60 cm x 30 cm x 30 cm and a water height of 20 cm are used for training the sticklebacks. The tanks are painted on all sides with commercially available blue dispersion paint to minimize disturbance of the fish by the experimenter or other factors. The tanks have two insertion options in which dividers (transparent and opaque) are inserted to obtain three compartments. Furthermore, each tank has two feeding tubes (25cm long, 2.5cm diameter) each with a lateral opening (approx. 1cm diameter) at the lower end of the pipe, which serve for the feed withdrawal. The tubes are inserted into a Plexiglas pane (30cm x 6cm, with two drill holes) and are placed in a corner of the aquarium. The pane has 2 markings which are painted on as squares (6cm edge length). These markings act as target zones later in the experiment.

*Fig. 1:* The training and experimental tank is divided into three compartments and the target zones (dotted lines in compartment 1) with the feeding tubes. The openings of the feeding tubes at the bottom face each other. The right compartment (3) is always delimited by a transparent divider plate (dashed line). The middle compartment (2) by an opaque (solid line).

All fish are trained twice a day for 15 days, whereby only the fish of one holding tank are transferred into the desired compartment of a test tank (e.g. 20 demonstrators of a holding tank into one of the test tanks). One training session takes 20 minutes, and the first 10 minutes is for adaptation. At the beginning of the second half, one of the feeders is provided with some chironomid larvae (location and color see table), the other with some drops of water from the thawed larvae and then the separating plate is removed. Sticklebacks are visual predators and the defrosted Chironomid water is added to rule out any olfactory prey. The sticklebacks are trained equally on both feeding tubes so that they cannot recognize any patterns during feeding. The distribution is random.

The demonstrators are placed in compartment 2 for acclimatization (see Fig. 1), after 10 min the opaque separation plate is removed and the sticklebacks start looking for food for another 10 min. Compartment 3 is therefore visible to the demonstrators, but they cannot explore this room at any time. Afterwards the separating plate is inserted again and the sticklebacks are transferred back into their holding tanks.

*Fig.2:* Experimental set-up in the demonstrators' training mode. The sticklebacks are placed in compartment 2 and receive a 10-minute acclimatization period. During this time, one feeding tube is provided with chironomid larvae and the other with the water of the defrosted larvae. After 10 minutes the opaque separating plate is removed and the fish may eat in the following 10 minutes.

The observers trained on green (ObsTrained) are initially placed in compartment 3. The procedure with the trained group of observers is the same as for the demonstrators. First, a 10-minute acclimatization phase is allowed and then again the search for food can begin. To do this, however, both separating plates must now be removed so that the sticklebacks can start their search of food.

*Fig. 3:* Experimental set-up in the training mode of the observers trained on green (ObsTrained). As already mentioned, the fish are placed in compartment 3 and after 10 minutes of acclimatization, the separating plates are removed.

The group of inexperienced sticklebacks is also trained (ObsUntrained). Here, however, the training only serves to get used to the test tank and to remove the separating plates. This group continues to receive the food in their respective holding tank during the 10 training days. Here, too, the group is transferred to compartment 3. After 10 minutes of acclimatization, both dividers are also removed, but there are no feeders left in compartment 1. This leaves the sticklebacks 10 minutes to explore the entire tank but not to feed.


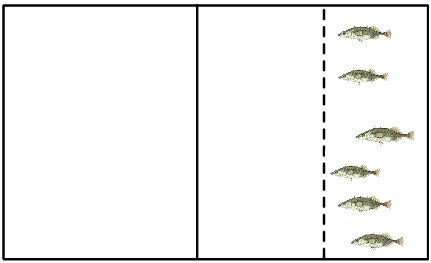


*Fig. 4:* Experimental setup in training mode of inexperienced observers without feeder (ObsUntrained). After 10 minutes of acclimatization, the separating plates are removed and the sticklebacks can explore the entire tank for another 10 minutes.

After 20 minutes of training, all separating plates are always inserted again and the sticklebacks are transferred back into their holding basins. After each training session, the test tanks are cleaned of remaining chironomid larvae. Furthermore, a 50 % water change is performed after each training day to completely clean the tanks of feces and remaining chironomid larvae.

Table for training

Demonstrator (Demo) and observer (ObsTrained): The position of the green feeding tube is indicated by the information on the left/right (randomly distributed). The color green or grey indicates into which tube the chironomids are filled, into the other only chironomid water.

Observer (ObsUntrained): No feeding tube in the tank and no food at all

S2 Experiments with sticklebacks by students – preparation script for students

Preparation script – Behavioral biology of fish

**The stickleback as a model organism for social aspects of food acquisition**

The test fish: The threespinedstickleback (*Gasterosteus aculeatus*) is found throughout Europe, Algeria, North Asia and North America, with the exception of the Danube Delta. It inhabits standing and flowing waters and lives both in fresh water and in coastal salt and brackish water. It is particularly suitable as an experimental animal because it is widespread, is not considered to be endangered and is very robust. The species is also easy to obtain, has hardly any demands on keeping and can be released back into the original waters after the experiments. In addition, the threespined stickleback is a popular object of behavioral research due to its interesting breeding behavior. The name threespined stickleback refers to the 3 spines that form the first part of the two-part dorsal fin.


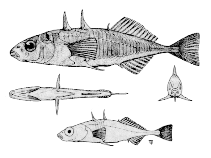

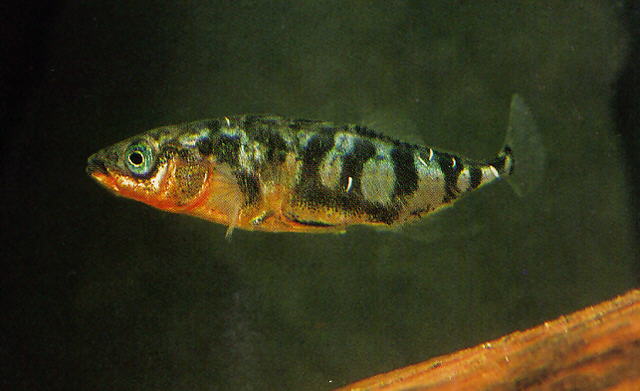


Threespined stickleback (*Gasterosteus aculeatus*) (left from: "Encyclopedia of Canadian Fishes" by Brian W. Coad, with Henry Waszczuk and Italo Labignan, 1995. right from: Brämick, U., U. Rothe, H. Schuhr, M. Tautenhahn, U. Thiel, C. Wolter and S. Zahn, 1998, Fische in Brandenburg.)

Like all sticklebacks (family of the Gasterosteidae) also the threespined stickleback does not carry scales. However, it forms overlapping bone plates like roof tiles, which are the distinguishing feature for three hereditary fixed variants:

forma trachurus (completely with bone plates)

forma semiarmatus (fragmentary with bone plates)

forma leiurus (with very low number of bone plates)

The extremely skilled swimmers feed on various small animals (insect larvae, worms) but also on fish spawn and brood. A pronounced sexual dimorphism in the threespined stickleback can only be seen during the breeding season, when the adult males wear a colorful breeding dress: The marbling retreats and the belly side turns intensely orange-red from the tip of the snout to the tail-stalk. The threespined stickleback shows a very special reproductive behavior in which the male builds a nest in which the eggs are laid according to a genetically determined pattern in the sequence of an action chain. A response chain is a sequence of individual instinctive actions, whereby each instinctive action of one partner triggers the next instinctive action of the other partner. Response chains run in the same defined sequence. Response chains often occur in reproductive behavior. The biological importance of a response chain lies in the avoidance of mismating between individuals of different species and in the local and temporal synchronization of the sexes during mating, so that fertilization is ensured. The spawning season is extremely stressful for both sexes and many sticklebacks die after their first spawning period. Outside of the reproductive-time, they live in loose swarms.

Experimental topic: Social learning corresponds to any event in which individuals acquire new behavior or information about the environment by observing or interacting with other animals (Brown & Laland, 2003). The experiment described here is based on experiments by Coolen et al. (2003) in which it was shown that sticklebacks are able to use clues from other individuals by observation to find food at a later date ("delayed local enhancement"). In our experiment we bring in an additional component, that of our own experience, in which we have trained some observers beforehand on a special feeding tube, before they are allowed to observe a group of sticklebacks in their search for food. A similar approach was already carried out with the 9-spined stickleback (*Pungitius pungitius*) (van Bergen et al. 2004, see appendix to the script). This could lead to a so-called "trade-off" situation in which the test fish must weigh up between their own learned knowledge ("private information") and the social information they have just seen ("public information").

Pretreatment of fish: We distinguish between the demonstrators and the two experimental groups of observers, the latter are actually the decisive experimental fish.

*Training Demonstrator:*

*Fig. 1:* The training and experimental tank is divided into three compartments and the target zones (dotted lines in compartment 1) with the feeding tubes. The openings of the feeding tubes at the bottom face each other. The right compartment (3) is always delimited by a transparent divider plate (dashed line), the middle compartment (2) by an opaque (solid line).

The demonstrators were trained twice a day for 15 days. One training session takes 20 minutes, and the first 10 minutes is for adaptation. At the beginning of the second half one of the feed pipes was provided with some chironomid larvae, the other with some drops of water from the defrosted larvae and afterwards the separating plate was removed. Sticklebacks are visual predators, but the thawed Chironomid water is added to rule out any olfactory prey. To prevent the sticklebacks from recognizing any patterns during feeding, they were trained equally on both feeding tubes (one green and one grey); the feeding tubes were randomly distributed to the right or left side of the tank. Also the addition of the food into the respective tube was finally coincidental. The demonstrators are thus trained to feed from one of the feeding tubes, regardless of its color and position in the aquarium.

*Training Observer:* There are two different groups of observers, one trained to feed from the green feeding tube (ObsTrained) and the other completely unexperienced (ObsUntrained). But this group is also being trained. Here, however, the training only serves to get used to the test tanks and to the removal of the separating plates. This group received their food during the 15 days of training from the surface in their respective holding tanks. Thus, there is a group of observers trained to feed from the green feeding tube and a second one which is completely inexperienced in this kind of feeding.

Experimental procedure:

Each group finds in its place an aquarium (60 cm x 30 cm x 30cm) which has 2 insertion possibilities and is prepared opaque from all sides. With the help of two separating plates (transparent and opaque) the aquarium can be divided into 3 compartments (see Fig. 1). The basin is also equipped with two feeding tubes (approx. 25 cm long, 2.5 cm diameter, one green and one grey), which can be attached at the ends to a Plexiglas plate (approx. 30 cm x 6 cm, with two drill holes) and each have an opening (approx. 1 cm diameter) at the ends, which serves for the food withdrawal. The feeding tubes are delimited by a ground marking, which should represent the target zones (edge length 6 cm). Furthermore, there is a mirror at one end of the tank, which is intended for the observation of the fish. This method of observation has already been proven, as the fish take little notice of the experimenter and show a natural behavior.

Prior to the experiment, a precise plan (based on a random distribution) is distributed, in which of the test aquariums which experiment is carried out. There are three test factors that are combined:

- Where to place the green feeding tube in the tank (right or left side)
- From which feeding tube demonstrators and observers are fed (green or grey)
- Whether the Observer was trained on green or is completely inexperienced (called untrained).

Please refer to the data sheet for your aquarium to find out which conditions have to be set for your test at your location.

Before the fish assigned to the groups are obtained from the holding tanks, the group's own test tank will be prepared as shown in Fig. 1, i.e. insert partitions, insert feeding tubes and install mirrors. The required fish can then be used. The demonstrators are always placed in compartment 2 and the observers always in compartment 3, so compartment 1 is initially concealed by the opaque separating plate for all fish.

*Fig 2:* Observation of the demonstrator. Top view of the test tank.

From now on it must be totally quieted in the course room and all students rely only on the commands of the lecturer. Once all the fish needed are in the test tank, a 10-minute acclimatization phase starts, during which the fish can acclimatize. Towards the end of the acclimatization phase, some defrosted chironomid larvae are placed in one of the feeding tubes (see your specific instructions for the pond) and some drops of the water of the defrosted larvae are placed in the other. Sticklebacks are visual predators, but some drops of water are added to completely exclude any possible olfactory prey. When the acclimatization phase ends, the opaque separating plate is removed and a 10-minute observation phase of the demonstrator begins (see Fig. 2). To synchronize all groups in the course room, a digital clock is presented and every action depends on the commands of the lecturer.

What is important now is the division within the group of students. It is advisable to divide the group into observers and timekeepers. First, the time of the "first hit" should be recorded, i.e. how much time (sec) has elapsed before the first stickleback reaches one of the target zones and whether its first choice was right or wrong. A stickleback is considered to be in the target zone if the pectoral fins exceed the mark. Subsequently, all feeding events are recorded on a tally chart. Picking after a larva is considered a feeding event, even if the stickleback didn’t swallow the food immediately. Furthermore, the number of sticklebacks in the right or wrong target zone at exactly this point in time is to be recorded in a defined 10-second rhythm (time-sampling) (also tally chart).

*Fig. 3:* Observation of the observer. Top view of the test tank.

After 10 minutes, the opaque plate is carefully placed directly in front of the transparent separating plate. Now the demonstrators are removed and transferred back into their holding tanks. The test tank and the feed tubes are quickly cleaned of chironomid larvae with a small landing net or under running water and filled with new larvae and their water as before. Now the separator plates are removed both in one action and the behavior of the observer is recorded for a further 5 minutes (see Fig. 3; same diagram, only one further parameter is recorded, namely when (sec) the right feeding area is reached for the first time).

At the end of the experiment, all fish are returned to the aquariums for intermediate storage, of course the demonstrator in the tank from which they were taken, and the observers as instructed by the instructors. Finally, the times/results of each individual fish must be entered in the prepared evaluation file. The evaluation is based on the following criteria:

- Demonstrator:

First decision to reach one of the target square (sec)
Was it the right (1) or wrong (2) square

Number of ticks per fish in the right or wrong target square
Number of ticks per fish for each ‘feeding event’

- Observer:

First decision to reach one of the target square (sec)
Was it the right (1) or wrong (2) square
If it was the wrong one, time (sec) until the right target square is reached
Number of ticks in the right or wrong target square
Number of ticks for each ‘feeding event’

After all values have been entered into the prepared file, we will analyze, statistically calculate and graph the data based on the hypotheses (what are they, think about it!).

For preparation:

In the appendix you will find an article (van Bergen et al. 2004) in which essential elements of the behavior of sticklebacks have been investigated in experiments similar to those covered in this lab. It is therefore imperative that the article is read before the lab starts. Furthermore, you should inform yourself about sticklebacks in general using e.g. Fishbase.org. The use of Excel and some basic statistical functions (e.g. t-test) is also preconditioned.

Further literature on the topic:

Brown, C.; Laland, K. (2002) Social learning of a novel avoidance task in the guppy, *P.reticulata*: Conformity and social release. Animal Behaviour, 64: 41-47.

Brown, C.; Laland, K. (2003) Social learning in fishes: A review. Fish and Fisheries, 4: 280-288.

Coolen, I.; van Bergen, Y.; Day, R.L.; Laland, K.N. (2003): Species difference in adaptive use of public information in sticklebacks. Biological Sciences. 270: 2413-2419

Day, R. MacDonald, T., Brown, C., Laland, K., and Reader, S.M. (2001) Interactions between shoal size and conformity in guppy social foraging. Animal Behaviour, 62: 917-925.

Honkanen, T.; Ekstrom, P. (1992): Comparative study of the olfactory epithelium of the three-spined stickleback (*Gasterosteus aculeatus*) and the nine-spined stickleback (*Pungitius pungitius*). Cell Tissue Res. 269: 267-273

Rowe, M.P.; Baube, C.L.; Phillips, J.B. (2006): Trying to see red through stickleback photoreceptors: Functional Substitution of Receptor Sensitivities. Ethology. 112: 218-229.

Swaney, W., Kendal, J., Capon, H., Brown, C. and Laland, K. (2001) Familiarity facilitates social learning of foraging behaviour in the guppy. Animal Behaviour, 62: 591-598.

van Bergen, Y.; Coolen, I.; Laland, K.N. (2004): Nine-spined sticklebacks exploit the most reliable source when public and private information conflict. Proc. R. Soc. Lond. B 271: 957–962
